# (±)-*trans*-3-Benzoyl­bicyclo­[2.2.2]octane-2-carboxylic acid

**DOI:** 10.1107/S1600536808024112

**Published:** 2008-08-06

**Authors:** Roger A. Lalancette, Hugh W. Thompson, Andrew P. J. Brunskill

**Affiliations:** a Carl A. Olson Memorial Laboratories, Department of Chemistry, Rutgers University, Newark, NJ 07102, USA

## Abstract

The title keto acid, C_16_H_18_O_3_, displays significant twisting of all three ethyl­ene bridges in its bicyclo­[2.2.2]octane structure owing to steric inter­actions; the bridgehead-to-bridgehead torsion angles are 13.14 (12), 13.14 (13) and 9.37 (13)°. The compound crystallizes as centrosymmetric carboxyl dimers [O⋯O = 2.6513 (12) Å and O—H⋯O = 178°], which have two orientations within the cell and contain no significant carboxyl disorder.

## Related literature

For related literature, see: Blackstock *et al.* (1987 ▶); Deutsch (1972 ▶); Scribner & Miller (1965 ▶); Zimmerman *et al.* (1992 ▶).
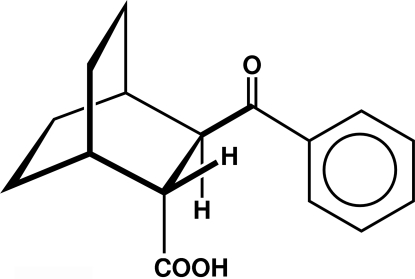


## Experimental

### 

#### Crystal data

C_16_H_18_O_3_
*M* *_r_* = 258.30
Monoclinic, 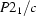

*a* = 7.9155 (7) Å
*b* = 11.1129 (9) Å
*c* = 14.7559 (12) Å
β = 93.882 (3)°
*V* = 1295.01 (19) Å^3^
*Z* = 4
Cu *K*α radiation
μ = 0.73 mm^−1^
*T* = 100 (2) K
0.49 × 0.30 × 0.17 mm

#### Data collection

Bruker SMART CCD APEXII area-detector diffractometer
Absorption correction: multi-scan (*SADABS*; Sheldrick, 2001 ▶) *T*_min_ = 0.716, *T*_max_ = 0.886
7983 measured reflections
2405 independent reflections
2350 reflections with *I* > 2σ(*I*)
*R*_int_ = 0.029

#### Refinement

*R*[*F* ^2^ > 2σ(*F* ^2^)] = 0.038
*wR*(*F* ^2^) = 0.100
*S* = 1.03
2405 reflections
174 parameters
H-atom parameters constrained
Δρ_max_ = 0.33 e Å^−3^
Δρ_min_ = −0.20 e Å^−3^

### 

Data collection: *APEX2* (Bruker, 2006 ▶); cell refinement: *APEX2*; data reduction: *SAINT* (Bruker, 2005 ▶); program(s) used to solve structure: *SHELXTL* (Sheldrick, 2008 ▶); program(s) used to refine structure: *SHELXTL*; molecular graphics: *SHELXTL*; software used to prepare material for publication: *SHELXTL*.

## Supplementary Material

Crystal structure: contains datablocks I, global. DOI: 10.1107/S1600536808024112/fl2213sup1.cif

Structure factors: contains datablocks I. DOI: 10.1107/S1600536808024112/fl2213Isup2.hkl

Additional supplementary materials:  crystallographic information; 3D view; checkCIF report

## Figures and Tables

**Table 1:** Hydrogen-bond geometry (Å, °)

*D*—H⋯*A*	*D*—H	H⋯*A*	*D*⋯*A*	*D*—H⋯*A*
O3—H3⋯O2^i^	0.84	1.81	2.6513 (12)	178

Symmetry code: (i) 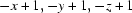.

**Fig. 1.**
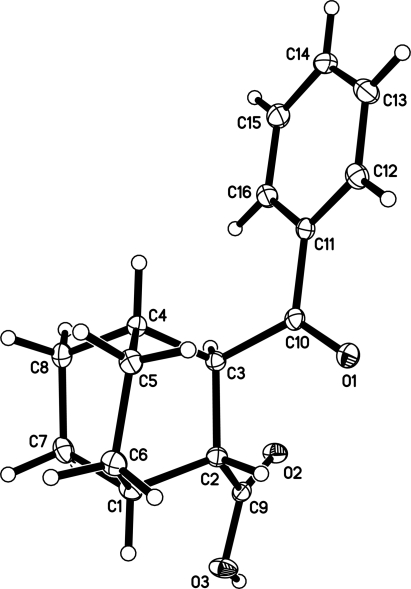
The asymmetric unit with its numbering. Displacement ellipsoids are drawn at the 40% probability level.

**Fig. 2.**
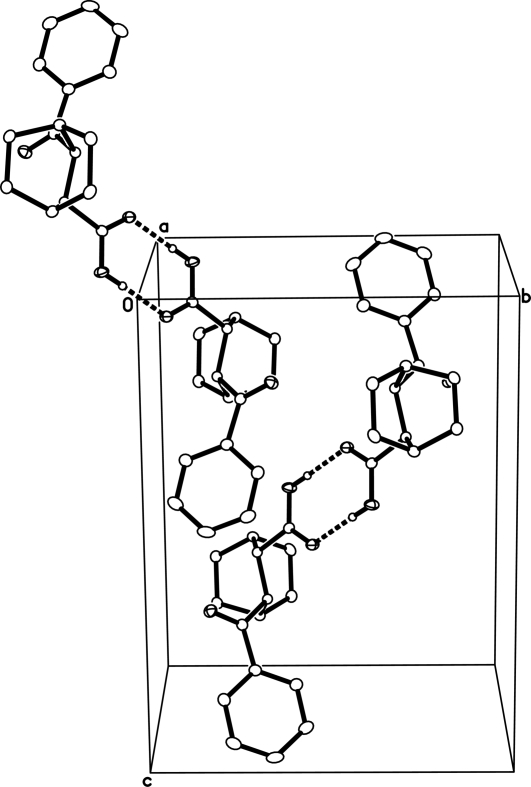
A partial packing diagram, with an extracellular molecule included to show the dimers centered at 1/2,1/2,1/2 and 1/2,0,0. For clarity, all C-bound H atoms are omitted. Displacement ellipsoids are drawn at the 40% probability level.

## supplementary crystallographic information

### Comment

Our study of crystalline keto acids concerns their repertoire of five known H-bonding modes. Two of these lack ketone involvement, including the commonest, acid dimerization, which is found in the aggregation of the title compound (I).

Fig. 1 shows the asymmetric unit of (I) with its numbering. Even simple bicyclo[2.2.2]octane systems often adopt a twist about the threefold axis, presumably to relieve eclipsing strain in their ethylene bridges (Deutsch, 1972; Blackstock *et al.*, 1987; Zimmerman *et al.*, 1992). In (I) that strain is supplemented by more serious eclipsing and 1,3-diaxial interactions involving the substituents, so that all three bridges are significantly twisted. Fig. 1 illustrates the extent to which the C5—C6 ethylene bridge is clearly not parallel to the others; however, the appearance of parallelism in the C2—C3 and C7—C8 bridges is an artifact of the viewing angle. The torsion angles for the three bridges are: C1—C2—C3—C4 = 13.14 (12)°, C1—C6—C5—C4 = 13.14 (13)°, C1—C7—C8—C4 = 9.37 (13)°.

The benzoyl group has component parts that are only approximately coplanar [the dihedral angle for C3—C10—C11—O1 *versus* the aromatic ring = 24.60 (7)°], and is oriented so that the ketone C=O is aimed toward C2. The carboxyl group is turned, with its C=O toward C3, so that the O2—C9—C2—C3 torsion angle is 18.76 (15)°. The dihedral angle between the ketone (C3—C10—C11—O1) and carboxyl group (C2—C9—O2—O3) is 79.80 (4)°. One may envision other possible conformations for the phenyl ring; however, because of steric hindrance, there is very little rotational freedom for the phenyl group here.

Although carboxyl dimers frequently display complete or partial averaging of C—O bond lengths and C—C—O angles due to disorder, no significant averaging is observed in (I), where these lengths and angles  are similar to those in other highly ordered dimeric carboxyls.

Fig. 2 shows the packing for (I), typical for racemic keto acids that are dimeric. Centrosymmetric dimers are centered at 1/2,1/2,1/2 in the chosen cell, with a second screw-related set centered on the a cell edge. No close intermolecular contacts were found within the 2.6 Å range we routinely survey for non-bonded C—H···O packing interactions.

### Experimental

*endo*-Bicyclo[2.2.2]oct-5-ene-2,3-dicarboxylic anhydride, purchased from Aldrich Chemical Co., Milwaukee, Wisconsin, USA, was hydrogenated under typical conditions (atmospheric pressure, room temperature, 5%Pd/C, EtOAc) and the isolated product used directly in a Friedel-Crafts acylation of benzene (AlCl_3_). The *cis* keto acid initially obtained (mp 446 K) was epimerized by refluxing in excess aqueous KOH (Scribner & Miller, 1965). The isolated *trans* product (I) was vacuum-distilled and crystallized from acetonitrile to give the crystal used, mp 444 K.

The solid-state IR spectrum (KBr) of (I) has C=O absorptions at 1692 (acid) and 1677 cm^-1^ (ketone), normal for dimerized COOH and for a benzoyl group without H bonding but with significant coplanarity. In CHCl_3_ solution these peaks appear at 1702 & 1679 cm^-1^.

### Refinement

All H atoms for (I) were found in electron density difference maps. The O—H was constrained to an idealized position with its distance fixed at 0.84 Å and *U*_iso_(H) = 1.5*U*_eq_(O). The methylene, methine and aromatic Hs were placed in geometrically idealized positions and constrained to ride on their parent C atoms with C—H distances of 0.99, 1.00 and 0.95 Å, respectively, and *U*_iso_(H) = 1.2*U*_eq_(C).

### Crystal data

**Table:**

C_16_H_18_O_3_	*F*_000_ = 552
*M**_r_* = 258.30	*D*_x_ = 1.325 Mg m^−^^3^
Monoclinic, *P*2_1_/*c*	Melting point: 444 K
Hall symbol: -P 2ybc	Cu *K*α radiation λ = 1.54178 Å
*a* = 7.9155 (7) Å	Cell parameters from 7078 reflections
*b* = 11.1129 (9) Å	θ = 3.0–69.4º
*c* = 14.7559 (12) Å	µ = 0.73 mm^−^^1^
β = 93.882 (3)º	*T* = 100 (2) K
*V* = 1295.01 (19) Å^3^	Block, colourless
*Z* = 4	0.49 × 0.30 × 0.17 mm

### Data collection

**Table:**

Bruker SMART CCD APEXII area-detector diffractometer	2405 independent reflections
Radiation source: fine-focus sealed tube	2350 reflections with *I* > 2σ(*I*)
Monochromator: graphite	*R*_int_ = 0.029
*T* = 100(2) K	θ_max_ = 69.8º
φ and ω scans	θ_min_ = 5.0º
Absorption correction: multi-scan(SADABS; Sheldrick, 2001)	*h* = −8→9
*T*_min_ = 0.716, *T*_max_ = 0.886	*k* = −13→11
7983 measured reflections	*l* = −16→17

### Refinement

**Table:**

Refinement on *F*^2^	Hydrogen site location: inferred from neighbouring sites
Least-squares matrix: full	H-atom parameters constrained
*R*[*F*^2^ > 2σ(*F*^2^)] = 0.038	*w* = 1/[σ^2^(*F*_o_^2^) + (0.0502*P*)^2^ + 0.6168*P*] where *P* = (*F*_o_^2^ + 2*F*_c_^2^)/3
*wR*(*F*^2^) = 0.100	(Δ/σ)_max_ < 0.001
*S* = 1.03	Δρ_max_ = 0.33 e Å^−^^3^
2405 reflections	Δρ_min_ = −0.20 e Å^−^^3^
174 parameters	Extinction correction: SHELXTL (Sheldrick, 2004), Fc^*^=kFc[1+0.001xFc^2^λ^3^/sin(2θ)]^-1/4^
Primary atom site location: structure-invariant direct methods	Extinction coefficient: 0.0033 (6)
Secondary atom site location: difference Fourier map	

### Special details

**Table:**

Experimental. crystal mounted on cryoloop using Paratone-N
Geometry. All e.s.d.'s (except the e.s.d. in the dihedral angle between two l.s. planes) are estimated using the full covariance matrix. The cell e.s.d.'s are taken into account individually in the estimation of e.s.d.'s in distances, angles and torsion angles; correlations between e.s.d.'s in cell parameters are only used when they are defined by crystal symmetry. An approximate (isotropic) treatment of cell e.s.d.'s is used for estimating e.s.d.'s involving l.s. planes.
Refinement. Refinement of *F*^2^ against ALL reflections. The weighted *R*-factor *wR* and goodness of fit *S* are based on *F*^2^, conventional *R*-factors *R* are based on *F*, with *F* set to zero for negative *F*^2^. The threshold expression of *F*^2^ > σ(*F*^2^) is used only for calculating *R*-factors(gt) *etc*. and is not relevant to the choice of reflections for refinement. *R*-factors based on *F*^2^ are statistically about twice as large as those based on *F*, and *R*- factors based on ALL data will be even larger.

### Fractional atomic coordinates and isotropic or equivalent isotropic displacement parameters (Å^2^)

**Table:**

	*x*	*y*	*z*	*U*_iso_*/*U*_eq_	
O1	0.61369 (11)	0.84095 (8)	0.26798 (6)	0.0210 (2)	
C1	0.15616 (14)	0.72901 (11)	0.34151 (8)	0.0160 (3)	
H1A	0.1178	0.7368	0.4044	0.019*	
O2	0.53283 (11)	0.55007 (8)	0.39852 (6)	0.0188 (2)	
C2	0.35047 (14)	0.71209 (10)	0.34504 (8)	0.0141 (3)	
H2A	0.4034	0.7906	0.3641	0.017*	
O3	0.33736 (12)	0.61674 (8)	0.48872 (6)	0.0219 (2)	
H3	0.3807	0.5638	0.5236	0.033*	
C3	0.40618 (14)	0.68316 (10)	0.24926 (7)	0.0135 (3)	
H3A	0.4327	0.5953	0.2465	0.016*	
C4	0.25626 (14)	0.71016 (10)	0.17863 (8)	0.0148 (3)	
H4A	0.2946	0.7028	0.1157	0.018*	
C5	0.19028 (15)	0.83791 (10)	0.19406 (8)	0.0169 (3)	
H5A	0.1055	0.8599	0.1444	0.020*	
H5B	0.2849	0.8961	0.1941	0.020*	
C6	0.10856 (15)	0.84268 (11)	0.28620 (8)	0.0181 (3)	
H6A	0.1487	0.9150	0.3204	0.022*	
H6B	−0.0161	0.8480	0.2758	0.022*	
C7	0.06974 (15)	0.62064 (11)	0.29348 (8)	0.0188 (3)	
H7A	−0.0546	0.6272	0.2959	0.023*	
H7B	0.1073	0.5453	0.3245	0.023*	
C8	0.11666 (15)	0.61758 (11)	0.19320 (8)	0.0180 (3)	
H8A	0.1567	0.5361	0.1780	0.022*	
H8B	0.0153	0.6361	0.1526	0.022*	
C9	0.41562 (14)	0.61797 (10)	0.41280 (8)	0.0147 (3)	
C10	0.56183 (14)	0.75308 (10)	0.22505 (8)	0.0147 (3)	
C11	0.64421 (14)	0.71562 (11)	0.14064 (8)	0.0154 (3)	
C12	0.73488 (15)	0.80248 (11)	0.09565 (8)	0.0191 (3)	
H12A	0.7467	0.8812	0.1204	0.023*	
C13	0.80759 (16)	0.77527 (12)	0.01549 (9)	0.0230 (3)	
H13A	0.8669	0.8356	−0.0152	0.028*	
C14	0.79381 (16)	0.65959 (13)	−0.02018 (9)	0.0245 (3)	
H14A	0.8438	0.6406	−0.0752	0.029*	
C15	0.70687 (16)	0.57201 (13)	0.02484 (9)	0.0246 (3)	
H15A	0.6996	0.4926	0.0011	0.030*	
C16	0.63028 (15)	0.59953 (11)	0.10437 (8)	0.0196 (3)	
H16A	0.5686	0.5396	0.1340	0.024*	

### Atomic displacement parameters (Å^2^)

**Table:**

	*U*^11^	*U*^22^	*U*^33^	*U*^12^	*U*^13^	*U*^23^
O1	0.0203 (4)	0.0220 (5)	0.0208 (4)	−0.0053 (3)	0.0021 (3)	−0.0048 (3)
C1	0.0145 (6)	0.0178 (6)	0.0162 (6)	0.0030 (4)	0.0036 (4)	0.0026 (4)
O2	0.0193 (4)	0.0220 (4)	0.0154 (4)	0.0064 (3)	0.0022 (3)	0.0017 (3)
C2	0.0148 (6)	0.0136 (5)	0.0141 (6)	0.0005 (4)	0.0015 (4)	−0.0004 (4)
O3	0.0241 (5)	0.0278 (5)	0.0141 (4)	0.0107 (4)	0.0039 (3)	0.0053 (3)
C3	0.0135 (5)	0.0135 (5)	0.0136 (5)	0.0009 (4)	0.0013 (4)	−0.0002 (4)
C4	0.0132 (5)	0.0168 (6)	0.0144 (5)	−0.0004 (4)	−0.0001 (4)	0.0002 (4)
C5	0.0161 (6)	0.0164 (6)	0.0181 (6)	0.0010 (4)	0.0005 (5)	0.0035 (4)
C6	0.0179 (6)	0.0166 (6)	0.0198 (6)	0.0044 (4)	0.0021 (5)	0.0012 (5)
C7	0.0147 (6)	0.0188 (6)	0.0230 (6)	−0.0016 (4)	0.0017 (5)	0.0052 (5)
C8	0.0150 (6)	0.0175 (6)	0.0214 (6)	−0.0018 (4)	−0.0003 (5)	−0.0014 (5)
C9	0.0146 (5)	0.0156 (6)	0.0137 (5)	−0.0005 (4)	−0.0007 (4)	−0.0017 (4)
C10	0.0128 (5)	0.0159 (6)	0.0150 (6)	0.0016 (4)	−0.0015 (4)	0.0015 (4)
C11	0.0106 (5)	0.0203 (6)	0.0152 (6)	0.0016 (4)	−0.0013 (4)	0.0012 (4)
C12	0.0158 (6)	0.0195 (6)	0.0219 (6)	0.0014 (5)	0.0012 (5)	0.0023 (5)
C13	0.0175 (6)	0.0298 (7)	0.0220 (6)	0.0012 (5)	0.0037 (5)	0.0069 (5)
C14	0.0179 (6)	0.0401 (8)	0.0157 (6)	−0.0002 (5)	0.0027 (5)	−0.0037 (5)
C15	0.0213 (6)	0.0291 (7)	0.0237 (6)	−0.0034 (5)	0.0032 (5)	−0.0094 (5)
C16	0.0167 (6)	0.0214 (6)	0.0209 (6)	−0.0025 (5)	0.0028 (5)	−0.0020 (5)

### Geometric parameters (Å, °)

**Table:**

O1—C10	1.2199 (15)	C6—H6A	0.9900
C1—C7	1.5346 (17)	C6—H6B	0.9900
C1—C6	1.5369 (16)	C7—C8	1.5503 (17)
C1—C2	1.5469 (15)	C7—H7A	0.9900
C1—H1A	1.0000	C7—H7B	0.9900
O2—C9	1.2251 (15)	C8—H8A	0.9900
C2—C9	1.5132 (16)	C8—H8B	0.9900
C2—C3	1.5426 (15)	C10—C11	1.5033 (16)
C2—H2A	1.0000	C11—C12	1.3971 (17)
O3—C9	1.3164 (14)	C11—C16	1.3982 (17)
O3—H3	0.8400	C12—C13	1.3835 (18)
C3—C10	1.5195 (15)	C12—H12A	0.9500
C3—C4	1.5547 (15)	C13—C14	1.391 (2)
C3—H3A	1.0000	C13—H13A	0.9500
C4—C5	1.5349 (16)	C14—C15	1.387 (2)
C4—C8	1.5356 (16)	C14—H14A	0.9500
C4—H4A	1.0000	C15—C16	1.3910 (17)
C5—C6	1.5456 (16)	C15—H15A	0.9500
C5—H5A	0.9900	C16—H16A	0.9500
C5—H5B	0.9900		
			
C7—C1—C6	108.31 (10)	C1—C7—C8	109.27 (9)
C7—C1—C2	109.35 (9)	C1—C7—H7A	109.8
C6—C1—C2	108.98 (9)	C8—C7—H7A	109.8
C7—C1—H1A	110.1	C1—C7—H7B	109.8
C6—C1—H1A	110.1	C8—C7—H7B	109.8
C2—C1—H1A	110.1	H7A—C7—H7B	108.3
C9—C2—C3	110.83 (9)	C4—C8—C7	109.89 (9)
C9—C2—C1	113.57 (9)	C4—C8—H8A	109.7
C3—C2—C1	109.86 (9)	C7—C8—H8A	109.7
C9—C2—H2A	107.4	C4—C8—H8B	109.7
C3—C2—H2A	107.4	C7—C8—H8B	109.7
C1—C2—H2A	107.4	H8A—C8—H8B	108.2
C9—O3—H3	109.5	O2—C9—O3	123.05 (10)
C10—C3—C2	113.29 (9)	O2—C9—C2	122.70 (10)
C10—C3—C4	109.75 (9)	O3—C9—C2	114.24 (9)
C2—C3—C4	108.75 (9)	O1—C10—C11	120.15 (10)
C10—C3—H3A	108.3	O1—C10—C3	122.48 (10)
C2—C3—H3A	108.3	C11—C10—C3	117.21 (10)
C4—C3—H3A	108.3	C12—C11—C16	119.04 (11)
C5—C4—C8	110.09 (9)	C12—C11—C10	117.90 (11)
C5—C4—C3	109.33 (9)	C16—C11—C10	123.04 (11)
C8—C4—C3	107.50 (9)	C13—C12—C11	120.80 (12)
C5—C4—H4A	110.0	C13—C12—H12A	119.6
C8—C4—H4A	110.0	C11—C12—H12A	119.6
C3—C4—H4A	110.0	C12—C13—C14	119.95 (12)
C4—C5—C6	109.22 (9)	C12—C13—H13A	120.0
C4—C5—H5A	109.8	C14—C13—H13A	120.0
C6—C5—H5A	109.8	C15—C14—C13	119.74 (12)
C4—C5—H5B	109.8	C15—C14—H14A	120.1
C6—C5—H5B	109.8	C13—C14—H14A	120.1
H5A—C5—H5B	108.3	C14—C15—C16	120.56 (12)
C1—C6—C5	109.68 (9)	C14—C15—H15A	119.7
C1—C6—H6A	109.7	C16—C15—H15A	119.7
C5—C6—H6A	109.7	C15—C16—C11	119.88 (12)
C1—C6—H6B	109.7	C15—C16—H16A	120.1
C5—C6—H6B	109.7	C11—C16—H16A	120.1
H6A—C6—H6B	108.2		
			
C7—C1—C2—C9	−73.31 (12)	C1—C7—C8—C4	9.37 (13)
C6—C1—C2—C9	168.48 (9)	C3—C2—C9—O2	18.76 (15)
C7—C1—C2—C3	51.44 (12)	C1—C2—C9—O2	142.99 (11)
C6—C1—C2—C3	−66.77 (12)	C3—C2—C9—O3	−162.26 (10)
C9—C2—C3—C10	−98.22 (11)	C1—C2—C9—O3	−38.03 (13)
C1—C2—C3—C10	135.46 (10)	C2—C3—C10—O1	−15.16 (15)
C9—C2—C3—C4	139.46 (9)	C4—C3—C10—O1	106.60 (12)
C1—C2—C3—C4	13.14 (12)	C2—C3—C10—C11	169.41 (9)
C10—C3—C4—C5	−72.95 (11)	C4—C3—C10—C11	−68.83 (12)
C2—C3—C4—C5	51.49 (12)	O1—C10—C11—C12	−22.30 (16)
C10—C3—C4—C8	167.56 (9)	C3—C10—C11—C12	153.24 (10)
C2—C3—C4—C8	−68.00 (11)	O1—C10—C11—C16	159.41 (12)
C8—C4—C5—C6	50.74 (12)	C3—C10—C11—C16	−25.06 (16)
C3—C4—C5—C6	−67.14 (11)	C16—C11—C12—C13	1.15 (18)
C7—C1—C6—C5	−67.71 (12)	C10—C11—C12—C13	−177.22 (11)
C2—C1—C6—C5	51.15 (12)	C11—C12—C13—C14	−1.35 (19)
C4—C5—C6—C1	13.14 (13)	C12—C13—C14—C15	0.09 (19)
C6—C1—C7—C8	54.02 (12)	C13—C14—C15—C16	1.4 (2)
C2—C1—C7—C8	−64.62 (12)	C14—C15—C16—C11	−1.54 (19)
C5—C4—C8—C7	−64.17 (12)	C12—C11—C16—C15	0.29 (18)
C3—C4—C8—C7	54.83 (12)	C10—C11—C16—C15	178.57 (11)

### Hydrogen-bond geometry (Å, °)

**Table:**

*D*—H···*A*	*D*—H	H···*A*	*D*···*A*	*D*—H···*A*
O3—H3···O2^i^	0.84	1.81	2.6513 (12)	178

Symmetry codes: (i) −*x*+1, −*y*+1, −*z*+1.
